# Influence of mudstone on coal spontaneous combustion characteristics and oxidation kinetics analysis

**DOI:** 10.1038/s41598-024-59797-8

**Published:** 2024-04-28

**Authors:** Xun Zhang, Jiahui Zou, Bing Lu, Gang Bai, Ling Qiao

**Affiliations:** 1 College of Mining, Liaoning Technical University, FuxinLiaoning, 123000 China; 2 College of Safety Science and Engineering, Liaoning Technical University, FuxinLiaoning, 123000 China; 3 Institute of Safety Engineering and Technology, Liaoning Technical University, FuxinLiaoning, 123000 China; 4 College of Safety and Emergency Management Engineering, Taiyuan University of Technology, Taiyuan, 030000 Shanxi China

**Keywords:** Coal spontaneous combustion, Mudstone, Pore structure, Oxidation characteristics, Thermodynamic properties, Fossil fuels, Coal

## Abstract

To explore the spontaneous combustion characteristics and hazards of the low-temperature oxidation (LTO) stage in the process of spontaneous combustion of coal and mudstone, the pore structure, spontaneous combustion characteristic parameters, and exothermic characteristics of coal and mudstone were tested and studied, and the oxidation kinetic parameters were calculated. The results show that mudstone has a larger specific surface area and pore volume than coal. From the fractal characteristics, the pore structure of mudstone is more complex than that of coal. According to the comparison of theoretical and actual gas generation and oxygen consumption rate curves, it is found that there is an interaction between coal and mudstone in the LTO process. With the increase of mudstone mass ratio, gas production, and its oxygen consumption rate increase. Among them, CM-4 (Coal:Mudstone = 1:1) has the highest exothermic intensity and the exothermic factor (A) and fire coefficient (K) increase with the increase of mudstone content. The apparent activation energy of the mudstone sample is lower than that of the raw coal, indicating that the sample after adding mudstone is more likely to have spontaneous combustion in the LTO stage.

## Introduction

Coal spontaneous combustion disaster in coal mine is not only a waste of resources but also a serious hazard to the health and safety of mine personnel^1–3^. Coal self-ignition is a dynamic and complex physicochemical process. The heat accumulated by the LTO of coal is the power source of coal self-ignition^4^. The essence of self-ignition of coal is a complex oxidation kinetic process in which coal and oxygen undergo physical and chemical reactions and release a large amount of heat and carbon monoxide equal gas. Therefore, carbon monoxide can be considered to be an indicator gas, and the characteristic changes of the coal self-ignition process can be analyzed by using the indicator gas method combined with the oxidation kinetic equation. The index gas method^5^ refers to selecting the index gas and analyzing its relationship with temperature. Many researchers domestic and abroad have studied the index gas method and the characteristic parameters in the course of coal self-ignition. Deng^6^ studied the characteristics of coal with divergent metamorphic degrees in the course of LTO and self-ignition by the temperature-programmed experiment and oxidation kinetics analysis. It was found that the stronger the grade-crossing-elimination structure of metamorphism of the coal, the greater the feature temperature and activation energy, and the lower the likelihood of coal samples spontaneously igniting. Yan^7^ studied the impact of pre-oxidation on the LTO of coal, studied the exothermic and kinetic characteristics of the LTO reaction, and discussed the influence of oxidation temperature and oxygen volume fraction on activating the following LTO response of pre-oxidized coal. The results show that the LTO process of pre-oxidized coal lags behind that of raw coal. Ren et al.^8^ studied the risk of self-ignition of pulverized coal and measured the LTO heat flow of three kinds of metamorphic pulverized coal at different levels of oxygen concentration by the C80 microcalorimeter system. The results indicate that the decrease in the density of oxygen and the enhancement in the metamorphic extent of pulverized coal greatly reduced the risk index of self-ignition of pulverized coal. Zhang^9^ studied the variation of organic sulfur compounds in coal during LTO and found that the gas release during LTO of SO_2_ is directly related to the metamorphic degree of coal and the content of the organic sulfur compound. Zhao^10^ studied the influence of water immersion on coal structure and LTO. It was found that the change in pore structure and the increase of free radical concentration caused by water soaking increased the possibility of contact among oxygen with active sites on the face of the pores, which made it more likely that the oxidation reaction would take place. Xiao^11^ studied the influence of ionic liquids on the macroscopic construction and critical temperature of LTO coal samples and concluded that ionic liquids would lead to a reduction in oxidation reactivity and the deterioration of oxidation performance. Dai^12^ studied the impact of oxygen supply on the LTO of coal and found that with the increase of oxygen concentration, the oxygen uptake rate and the yield of gaseous products increased to a certain extent. Zhong^13^ studied the heat effect of low-rank coal in the process of LTO after immersion in water and vacuum drying. It was found that the fast aliphatic chain-forming reaction was promoted after immersion in water and vacuum drying, which increased the initial heat release and made the coal more prone to self-ignition. Zhang^14^ studied the influence of long-term immersion in water on the LTO process of coal and concluded that long-term immersion led to significant periodic changes in the oxidation property of coal, of which 6 months of immersion time was crucial.

In the process of LTO of coal, lateral chain and various oxidized functional groups mainly participate in the reaction. The functional group is slowly activated and begins to react with oxygen. Among the many oxidized functional groups in coal, the hydroxyl group is the key active substance to induce the LTO of coal^15^, and the carbonyl group is considered to be helpful to induce the transformation of chemical adsorption to LTO^16^. Li et al.^17^ used a combination of LTO and low-temperature carbonization to determine the macro gas concentration of the two processes and then used in-situ infrared spectroscopy and in-situ EPR to determine the content of microscopic free radicals and oxygen-containing functional groups. It was discovered that low-temperature pyrolysis led to the degradation of oxidized functional groups and the creation of alkyl radicals. The thermal cracking of oxidized functional groups generates many active sites, which are oxidized at room temperature to release mass gas and release heat^18^. Meng^19^ studied the formation, release, and changes of active radicals of small molecular gases in low-rank coal during LTO by TG-DSC, TG-IR, and in-situ infrared measurements, and found that 100–200 °C is the key temperature for gas production. Zhang^20^ studied the impact of radicals on heat during the influence of long-term immersion of bituminous coal was studied. The results showed that the hydroxyl content increased with the increase of the grade-crossing-elimination structure of metamorphism, and the key functional groups of bituminous coal played an important role in heat.

Coal inevitably undergoes various changes in its physical structure and surface morphology during LTO^21^, which affects the heat and mass transfer efficiency when oxidizing coal at low temperatures^22^. To illustrate, the micropores of the coal affect the maximum heat release and the initial heat release of LTO^23^. During the LTO of coal, many mesoporous channels are formed. These provide a physical space for oxygen migration and adsorption^24^. In the process of LTO, in general, the amount of oxygen adsorbed on the coal surface is affected by the growth of the pore structure. Cai et al.^25^ studied the surface physical microstructure evolution of different metamorphic coals during LTO and explained the influence of coal surface texture on oxidizing property through pore connectivity and oxygen adsorption on coal surface during coal oxygen reaction.

At present, the research on low-temperature oxidation (LTO) of coal has been relatively perfect, but the research on LTO of mudstone and coal blending is relatively scarce. In fact, in the process of coal mining, the roof mudstone will collapse and mix with coal in the goaf. Therefore, in this paper, the temperature-programmed experiment and low-temperature nitrogen adsorption experiment were used to test the LTO process of coal-rock mixtures with different mass ratios. The specific surface area, pore volume, pore size distribution, and fractal characteristics of coal and mudstone were compared, and the changes in index gas, oxygen consumption rate, exothermic intensity, oxidation kinetic parameters, and other parameters in the LTO process of coal and mudstone were discussed. The research results are of great significance to the spontaneous combustion characteristics of the coal-rock mixture in an LTO process in Goaf.

## Materials and methods

### Samples preparation

The test coal samples were taken from Pingzhuang lignite in Inner Mongolia, and the rock samples were taken from the mudstone of the coal seam roof. After sampling, it was sealed under a vacuum and taken to the laboratory. The lignite and mudstone were ground to 100–150 mesh with a jaw crusher. The coal sample and the rock sample were mixed according to the mass percentage ratio of 1:0, 1:0.1, 1:0.4, 1:0.7, 1:1 and 0:1, Named C, CM-1, CM-2, CM-3, CM-4, M, vacuum drying at room temperature for 24 h, sealed and stored. The industrial analysis of coal and mudstone is shown in Table 1.

**Table 1:** Proximate analysis of coal and mudstone.

Sample	M_t_/%	A_ad_/%	V_ad_/%	FC_ad_/%
Coal	9.17	12.37	32.91	45.55
Mudstone	2.79	87.33	8.25	1.63

### Experiments

#### Specific surface determination

The pore distribution of coal and mudstone samples was measured by BELSORP-maxII specific surface area and pore size distribution analyzer of Macchibay, Japan. The test sample mass is approximately 1 g. This was followed by the calculation of specific surface area and pore distribution using BET multilayer adsorption theory and BJH.

#### Temperature programmed gas production experiment

**Figure 1:**
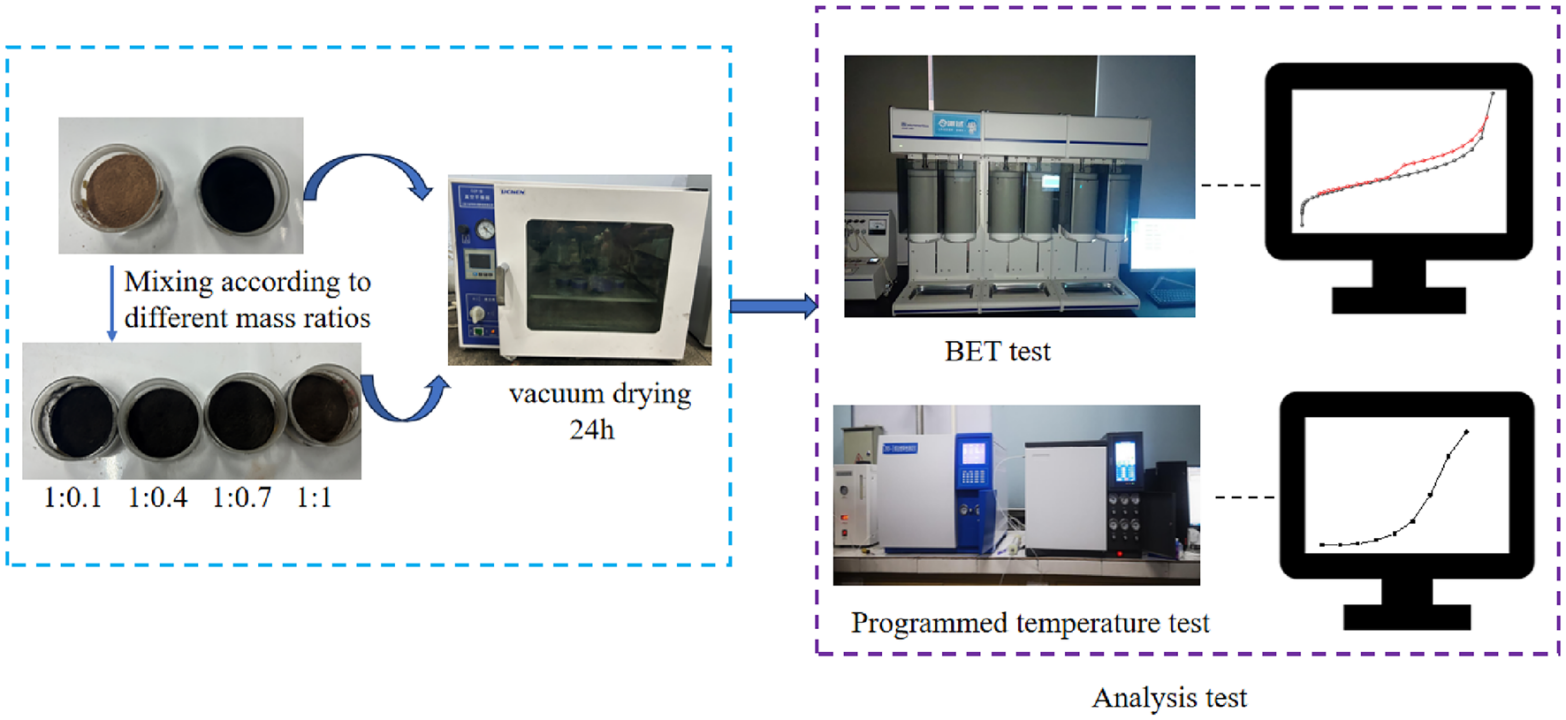
Flow chart of experiment.

The ZRD-II coal spontaneous combustion characteristic tester and HW-2000 chromatograph combined system were used to take 10 g coal-rock mixed samples. The mixed coal sample was placed in a coal sample tank for a temperature rise test. Fresh air (oxygen volume fraction of 20.95%) was introduced, the flow rate was 30 mL/min, and the temperature was increased from 40 to 200 °C. When the coal temperature reached the temperature point to be measured, the air pump was opened to pass the gas into the chromatograph, and the gas analysis and recording were completed in the computer terminal software. The experimental equipment and steps are shown in Fig. 1.

## Results and discussions

### Pore structure analysis

#### Low temperature N_2_ adsorption test results

**Figure 2:**
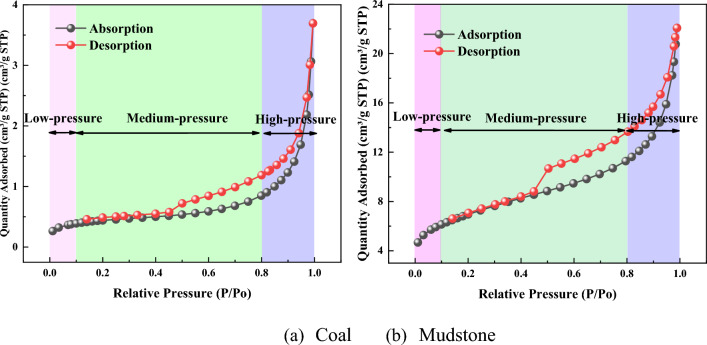
Adsorption/desorption curves of lignite and mudstone. (**a**) Coal. (**b**) Mudstone.

Figure 2 shows the low-temperature N_2_ adsorption/desorption isotherms of coal and mudstone. The N_2_ adsorption/desorption isotherms show a significant hysteresis loop in the range of P / P_0_ between 0.45 and 1 and are classified as type IV or IV (a), which is strongly linked to the capillary condensation in the mesopores^26^. At about P/P_0_ = 0.5 the steep region of the desorption isotherm leads to the lower closing point of the hysteresis loop. Monolayer and multilayer adsorption, dominated by van der Waals forces, produces the initial part before the lower closure point^27^. The hysteresis curve of coal and mudstone belongs to the H3 type, representing slit-like pores^28^, indicating that the pores are in a half-open position. Compared with coal, mudstone has a more obvious hysteresis loop. The more apparent the hysteresis effect, the worse the pore network connectivity, which also suggests that there are a large number of irregular nanopores in mudstone. It is worth noting that when the relative pressure is 0.5, there is a mutation point in the desorption branch, which represents the minimum value of pore development^29^. The amount of nitrogen adsorbed in the studied samples varies greatly, while mudstone adsorbs more nitrogen at the highest relative pressure (about 22.078 cm^3^/g), showing greater adsorption capacity, while coal adsorbs less nitrogen (about 3.695 cm^3^/g). According to the characteristics of the adsorption, the isothermal adsorption curve can be partitioned into three stages, stage I (P/P_0_ < 0.1), stage II (P/P_0_ = 0.1–0.8) and stage III (P/P_0_ = 0.8–1). The first stage is the low-pressure stage. The gas adsorption in this stage mainly exists in the form of single-layer adsorption. Due to the low micropore content, the adsorption capacity of coal and mudstone is low, and the adsorption curve rises slowly. With the increase of relative pressure, the adsorption of a single layer is completed. Entering the intermediate pressure stage, the power-level trip of adsorption of coal and mudstone increases with the increase of relative static pressure, but the rate of increase of the two is different. The power-level trip of adsorption of coal began to augment when the relative static pressure reached about 0.6, while the power-level trip of adsorption of mudstone continued to increase during the medium pressure process. Within this relative pressure enclosure, with the increase of relative static pressure, the adsorption of N_2_ in coal pores moderately changes from single-layer adsorption to polymolecular layer adsorption on larger borehole wall (Pores capable of accommodating at least two layers of nitrogen). If the relationship between relative pressure and pore width satisfies the Kelvin equation, capillary condensation occurs in the pores^30^. With the increase of pressure, the N_2_ molecule will undergo agglomeration in larger pores. The third stage is the high-pressure stage, and the sorptive capacity of coal and mudstone increases exponentially in this area. This indicates that there is massive liquefaction of N_2_ in the macro pores or on the faces of the sample^31^, resulting in a dramatic increase in N_2_ sorptive ability. Even at relative pressures near 1, there is still no attachment threshold. Pay attention, the sorptive capacity of mudstone has kept a greater growth rate throughout the process. This suggests that there are pores of appropriate size at any relative pressure band. Therefore, the pore distribution of mudstone is more homogeneous than that of coal samples. This means that the adsorption ability of the sample is superior to that of the coal used.

#### Pore structure evolution

Table 2 gives the pore structure parameters of coal and mudstone. It can be seen that the specific surface area of mudstone is 21.18 times that of coal. Mudstone has a larger specific surface area than coal, so mudstone can adsorb more gas. The total pore volume of mudstone is 5.98 times that of coal, signifying that the porous microstructure of mudstone is more developed, with more mesopores and macropores.

**Table 2:** Specific surface area and pore structure parameters of coal and mudstone.

Coal sample	Specific surface area (m^2^/g)	Total pore volume (cm^3^/g)	Average pore size (nm)
M	1.16118	0.005715	14.1829
Y	24.9520	0.034151	5.4746

**Figure 3:**
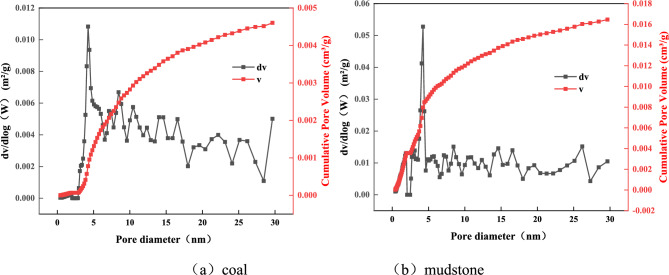
PSDs of coal and mudstone (pore size less than 30 nm). (**a**) Coal. (**b**) Mudstone.

Density functional theory was used to analyze the adsorption isotherm curves of coal samples, and the pore size distribution of coal samples in the range of 0–30 nm was obtained. The red curve in Fig. 3 reflects the variation in cumulative pore volume, and the black curve shows the variation rate of increase of pore space volume with pore size. The broad peak indicates that the pore size of the interval in question is a complex one, and the narrower peak means that the pore size of the interval is fairly uniform. See Fig. 3, when the pore size is under 2 nm, the v, and dv of the coal sample are close to 0, indicating that it contains a small amount of micropores. There is a strong peak at 2–5 nm in both coal and mudstone, signifying that there are more pores in the range of 2–5 nm, and the pore size is concentrated at about 4.2 nm. In the range of 5–30 nm, there are many peaks of different sizes, indicating that coal and mudstone distribute many pores with uneven pore size in this range.

**Figure 4:**
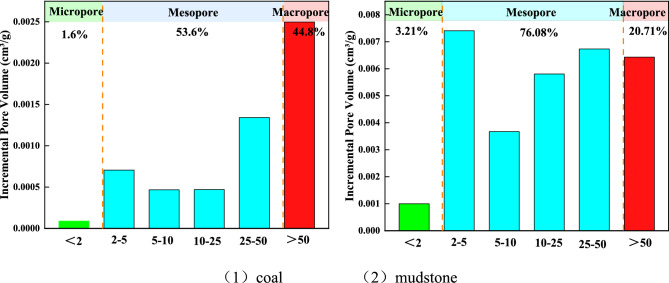
Coal and mudstone pore volume increment. (1) Coal. (2) Mudstone.

The BJH method was used to examine the adsorption isotherms, and the pore volume and pore percentage in the range of 0–150 nm were obtained. It can be seen from Fig. 4 that the pore volume of coal and mudstone is mainly provided by mesopores, accounting for 53.6% and 76.08% of the total pore volume, respectively, followed by macropores, accounting for 44.8% and 20.71% of the total pore volume, respectively, and micropores account for the least. Within the pore volume of 30 nm, the pore volume at 2–5 nm accounts for the largest proportion, which corresponds to the strong peak at 2–5 nm in Fig. 3.

#### Fractal dimension calculation

The fractal dimension (Ds)^29,32^ is widely used to represent the surface complexity and smoothness of holes. Studies have shown that in the process of adsorption, desorption, and diffusion, an important role is played by the irregularity of the pore surface. The Frenkel–Halsey–Hill (FHH) model was used to analyze the N_2_ isothermal adsorption/desorption process of coal. Compared to other models, the FHH model is the most effective^33^.

**Figure 5:**
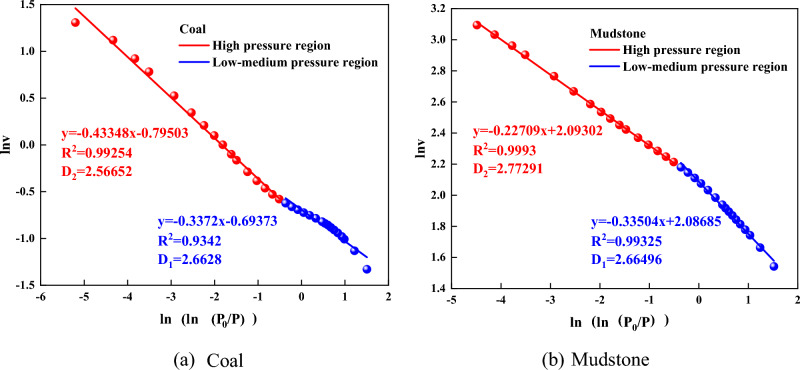
FHH model calculation results. (**a**) Coal. (**b**) Mudstone.

**Figure 6:**
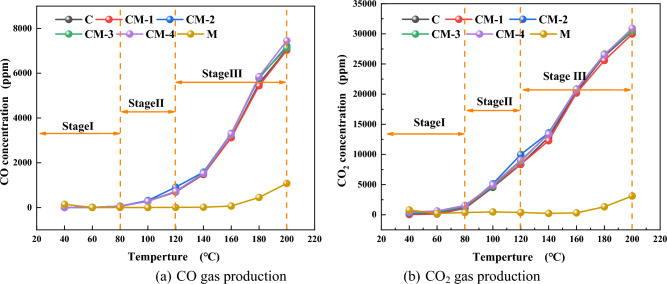
Analysis of CO and CO_2_ production of coal and mudstone and coal-rock mixtures with different mass ratios. (**a**) CO gas production. (**b**) CO_2_ gas production.

Based on the data from the gas adsorption diagram, the FHH diagram can be generated (see Fig. 5). As a general rule, the FHH curve has a closure point at P/P0 = 0.5. The curve is split into two parts, and the fractal number of dimension Ds of the two parts is denoted by D_1_ and D_2_, respectively. D_1_ is the surface roughness characterization of pores, and D_2_ is a characterization of the complexity of the pore structure. The higher the D_1_ value, the coarser the surface of the pores; the higher the D_2_ value, the more complex the structure of the pores. As shown in Fig. 5, the fractal characteristics of mudstone and coal, in which the greater the coefficient of association R^2^, the more meaningful the fractal properties. It can be seen from Fig. 6 that the D_1_ of coal and mudstone is the same, indicating that the roughness of the pore surface of the two is similar, but the D_2_ of the two is very different. The D_2_ of mudstone is 0.20594 greater than coal, signifying that the pore structure of mudstone is more complex than that of coal, which is reflected in the pore size distribution of Fig. 3. The fractal dimension of mudstone samples is greater than 2.6, indicating that the surface roughness is high, the pore connectivity is poor, and the reservoir heterogeneity is strong^34^. Among them, mudstone D_2_ > D_1_, reflecting the heterogeneity of mudstone pore structure is slightly stronger than pore surface roughness and micropore irregularity. Studies have shown that^35^, when the complexity of the pore structure of macropores is greater than that of micropores, D_2_ will be greater than D_1_. The reason is that the types of macropores are diverse, including intergranular pores, intragranular pores, intercrystalline pores, etc., while the types of micropores are relatively simple, so the macropore structure is more complex. It has a fairly loose spatial structure, which is similar to the study of Zhao et al.^36^. The coal D_1_ > D_2_ indicates that the irregularities and disturbances of the coal surface are more sturdy than the irregularity and disorder of the pore structure inside the coal. Similar to adsorption performance and pore structure properties.

In summary, coal and mudstone are complex porous materials, and these pores affect adsorption, desorption, diffusion, and seepage. The pore size, pore volume, and specific surface area of coal and mudstone affect their oxygen absorption capacity, which in turn affects the spontaneous combustion characteristics of coal.

### Experimental analysis of temperature programming

#### Analysis of iconic gas production

Coal spontaneous combustion is a slow process, the oxidation process will produce different gas products, such as carbon monoxide, carbon dioxide, and other gases. Carbon monoxide gas is the main product of the photooxidation reaction of coal. In contrast to other gaseous products, CO gas is produced when coal begins to spontaneously combust, which bodes well for spontaneous coal burning.

Figure 6a shows the variation of carbon monoxide and carbon dioxide production with temperature in coal-mudstone and coal-rock mixtures with different proportions, while Fig. 6b shows the trend in CO_2_ production with temperature. According to the data analysis, the changing trend of CO_2_ and CO curves of coal and coal-rock mixture is consistent. In the LTO stage, the production of carbon monoxide, and carbon dioxide is gradually increasing, showing an exponential growth trend. According to the gas growth trend, the oxidation at the ambient temperature curve is divided into three stages, and the specific division details are given in the figure. In the first stage, because the temperature is not fully accumulated, the CO yield of the sample remains unchanged, so the oxidation reaction remains slow. However, a small amount of CO and CO_2_ gas is released from mudstone before 60 °C. The reason is that the specific surface area of mudstone is larger than that of coal, and the secondary CO in the free state of pores begins to desorb^37^, this leads to relatively high CO and CO_2_ production at this stage. As temperature rises, entering the second stage, the active groups on the surface of coal begin to chemically adsorb and the active sites generated during the heating process begin to react with oxygen to produce CO, which makes the CO generation of coal and samples added with mudstone began to increase. At this stage, the gas production of mudstone showed a steady trend. At this stage, the mudstone had insufficient aromatic and aliphatic compounds^38^. As a result, it will not undergo an oxidation reaction in this process. In the third stage, the reaction between the coal and the oxygen is very intense, and the CO and CO_2_ gases increase exponentially and enter the rapid oxidation stage. The CO and CO_2_ production of mudstone begins to increase slightly after 160 °C, and the low content of carbon is oxidized in this process. The chemical adsorption of mudstone begins to produce a small amount of CO and CO_2_ gases.

In summary, during the LTO process of coal-rock mixtures with different mass ratios, the gas production curve is similar to raw coal. The CO and CO_2_ gas concentrations of coal-rock mixtures are more than those of raw coal and mudstone, but the increase is not large. It shows that there is a certain interaction between mudstone and LTO of coal.

#### Oxygen consumption rate analysis

**Figure 7:**
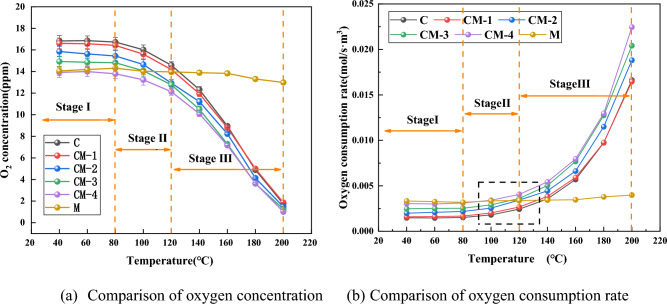
Comparison of oxygen concentration and oxygen consumption rate of coal and mudstone and coal-rock mixtures with different mass ratios. (**a**) Comparison of oxygen concentration. (**b**) Comparison of oxygen consumption rate.

**Figure 8:**
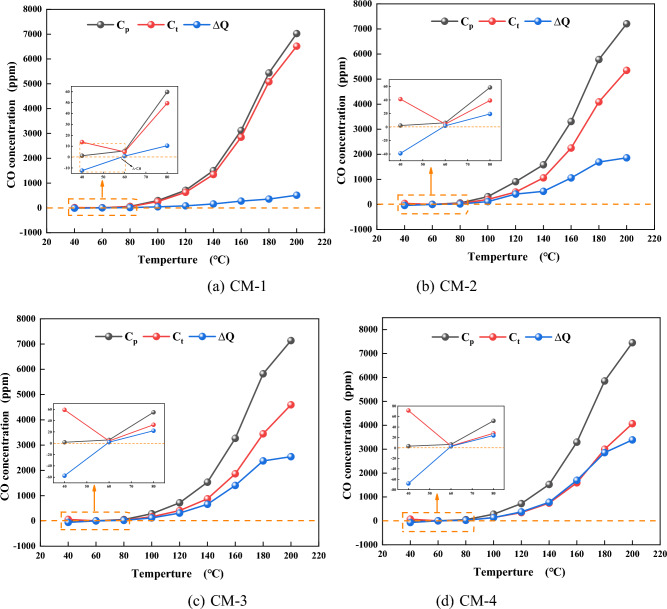
Changes of actual CO production and theoretical CO production during LTO of coal-rock mixtures with different proportions. (**a**) CM-1. (**b**) CM-2. (**c**) CM-3. (**d**) CM-4.

The theory of coal-oxygen reaction has revealed the microscopic mechanism of coal oxidation^41,42^. Oxygen is the dominant factor of the coal-oxygen response. Figure 7 is the comparison of oxygen consumption rates of coal-rock mixtures with alternative mass ratios and coal and mudstone. The oxygen concentrations of coal and mudstone and coal-rock mixtures with different mass ratios are shown in Fig. 8a. The oxygen concentration gradually decreases as the temperature increases, as can be seen from the figure, and the decrease is more evident with the rise of mudstone content. See Fig. 8b, in stage I, due to the low level of coal oxidation, the variation in oxygen consumption rate is not obvious, and the corresponding oxygen consumption rate^39^ is low. The samples of coal and mudstone are consumed in a small amount of oxygen in the first stage. At this point, physical adsorption is the main controlling factor of the coal-oxygen responses. The intensity of the physical adsorption reaction is relatively weak, resulting in a gentle increase in the standard oxygen consumption of coal. At this time, there is a large amount of oxygen consumption in mudstone and the consumption remains stable in the first stage. The reason is that mudstone has a large specific surface area and a relatively developed pore structure, and a large amount of oxygen is adsorbed during this period. As coal temperature increases, the number of reactive functional groups and active sites increases, especially in stage II and stage III, the highly absorbent substances in mudstone promote the vaporization of coal water^40^, more active sites begin to be exposed, and the rate of oxidation of the coal is accelerated, which in turn accelerates the oxygen consumption velocity. As the volume of mudstone added increases, the oxygen demand rate of the sample increases, indicating that the addition of mudstone accelerates the chemical oxygen of the mixed sample. The chemical oxygen rate of all samples except mudstone shows an upward trend. Through industrial analysis, it is possible to see that the lower carbon content and its lower volatile matter in mudstone are related to its difficult oxidation reaction^41^.

#### Transactional analysis

To investigate the mechanism of mudstone and coal in the ambient temperature oxidation stage, the experimental study of coal and mudstone was carried out. The theoretical gas production curve C_t_ and the theoretical oxygen demand rate curve O_t_ of the coal and mudstone mixture were calculated by the equation model. The difference between the gas production curve ΔQ and the difference between the oxygen consumption rate curve ΔH were calculated by the equation. The calculation method is shown in Eqs. (1)–(4):

1 $${C}_{{\text{t}}}={x}_{{\text{m}}}{C}_{{\text{m}}}+{x}_{{\text{y}}}{C}_{{\text{y}}}$$

2 $${O}_{{\text{t}}}={x}_{{\text{m}}}{O}_{{\text{m}}}+{x}_{{\text{y}}}{O}_{{\text{y}}}$$

3 $$\Delta Q={C}_{{\text{p}}}-{C}_{{\text{t}}}$$

4 $$\Delta H={O}_{{\text{p}}}-{O}_{{\text{t}}}$$

where ${x}_{{\text{m}}}$, ${x}_{{\text{y}}}$ is the mass fraction of coal and mudstone, %; ${C}_{{\text{m}}}$, ${C}_{{\text{y}}}$ is CO gas release from coal and mudstone, ppm; ${O}_{{\text{m}}}$, ${O}_{{\text{y}}}$ is oxygen consumption rate of coal and mudstone, %.

**Figure 9:**
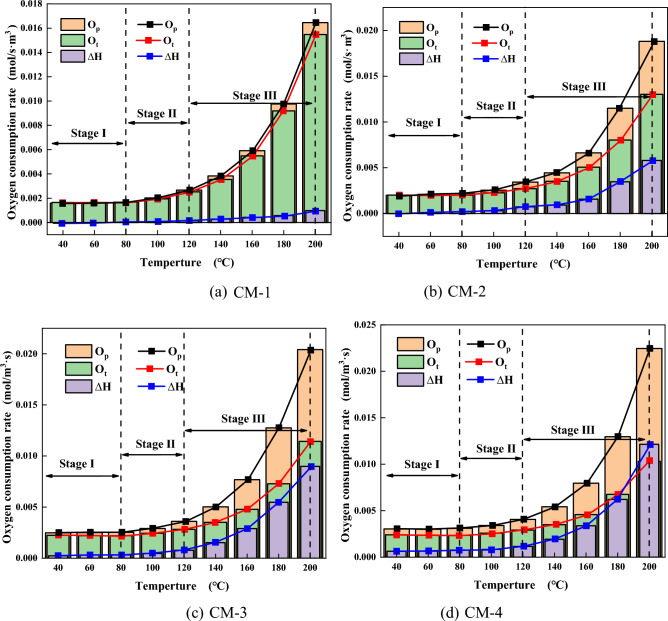
Changes of actual oxygen consumption rate and theoretical oxygen consumption rate during LTO of coal-rock mixtures with different proportions. (**a**) CM-1. (**b**) CM-2. (**c**) CM-3. (**d**) CM-4.

**Figure 10:**
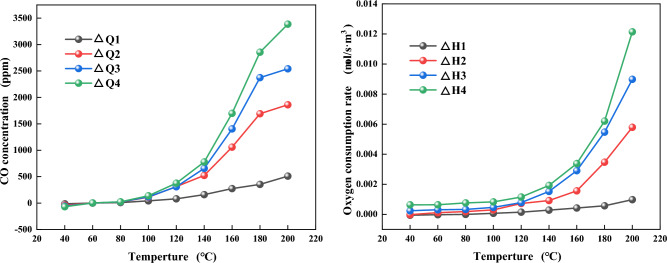
Changes of ∆Q and ∆H of each sample.

The changes of ΔQ and ΔH in the course of LTO of coal and mudstone under different mass ratios are compared as shown in Figs. 8 and 9. The results show that mudstone samples with different mass ratios have some impact on the interaction in the process of LTO. It is visible from Fig. 8a that when the temperature is less than 60 °C, ΔQ0, there is a mutual inhibition effect between coal with rock at this stage. The strong absorbent minerals illite and montmorillonite in mudstone absorb the water in coal^42^. The free water is covered on the surface of the sample and enters the pores, which hinders the gas desorption of the sample so that the actual gas release of the mixed sample is lower than the theoretical gas release at this stage. At 60–200 °C, ∆Q and ∆H are greater than 0. It can be seen from the variation of ∆Q and ∆H of each sample in Fig. 10 that ∆Q and ∆H gradually rise with the increase in silt content, indicating that there is a mutual promotion effect between mudstone and coal at this stage, and the greater the mudstone content is, the more pronounced the promotional impact. The reason is that the higher sulfur content and mineral content in mudstone plays a catalytic part in the oxidation reaction of the sample^43^. The additive of mudstone accelerates the oxygen consumption of the mixed sample. The larger specific surface area of mudstone adsorbs a large amount of oxygen. The more mudstone provides more adsorbed oxygen, which provides an oxygen supply channel for coal oxidation. The oxygen consumption increases significantly, the functional groups in coal are further active, and active sites are continuously generated^44^. A steady stream of oxygen molecules is adsorbed on these active sites, resulting in an increase in CO and CO_2_ gas production. It shows that in the procedure for LTO, mudstone has played a certain role in promoting coal spontaneous combustion.

### Analysis of heat release intensity

#### Exothermicity

Coal spontaneous combustion is a phenomenon caused by the continuous rise of coal temperature due to the photooxidation reaction of coal with oxygen in the air and the release of a huge quantity of reaction heat. Therefore, in the combustion of coal, the exothermicity of coal can characterize the oxidation process of coal at a certain temperature point or a certain temperature range. As an important index to measure the heat release of coal, the calculation and analysis of heat release intensity is indispensable. According to the change of bond energy, the calculation formula of exothermic intensity is estimated. See Eq. (5):

5 $${q}_{\text{max}}(T)=\Delta {H}_{20}[{v}_{{{\text{o}}}_{2}}(T)-{v}_{{\text{co}}}(T)-{v}_{{{\text{co}}}_{2}}(T)]+\Delta {H}_{{\text{co}}}{v}_{{\text{co}}}(T)+\Delta {H}_{{{\text{co}}}_{2}}{v}_{{{\text{co}}}_{2}}(T)$$

where, ${q}_{\text{max}}$ is the maximum exothermic intensity, J/(cm^3^ s); $T$ is the thermal temperature of coal, K; $\Delta {H}_{20}$ is the average heat of the second step, 284.97 kJ/mol; ${v}_{{{\text{o}}}_{2}}$ is oxygen consumption rate, mol/(cm^3^ s); ${v}_{co}$ is the CO production rate, mol/(cm^3^ s); ${v}_{{{\text{co}}}_{2}}$ is the rate of CO_2_ production, mol/(cm^3^ s); the average reaction heat of $\Delta {H}_{{\text{co}}}$ to 1 mol CO, 311.9 kJ/mol; $\Delta {H}_{{{\text{co}}}_{2}}$ is the average reaction heat of generating 1 mol CO_2_, which is 466.7 kJ/mol.

**Figure 11:**
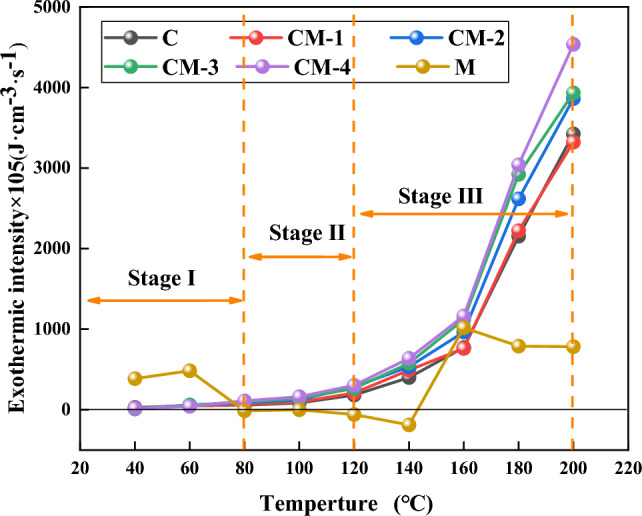
Comparison of heat release intensity.

It can be seen from Fig. 11 that the exothermic intensity of coal and coal-rock mixtures with different proportions increases with the temperature rise. In the three stages of LTO, the oxidation reaction intensity of the samples is different. In stage I, only a small quantity of easily oxidized primary active groups participate in the reaction, create secondary groups of active users, and release a small amount of heat. At this time, the addition of mudstone has only a minor impact on the heat release of the sample. In stage II and stage III, with the rise of mudstone content, the heat release intensity shows an increasing trend. The highest intensity of the thermal output of CM-4 is 4533.9 × 10^5^ (J cm^−3^ s^−1^), which is 1.33 times that of raw coal. On the one hand, mudstone provides sufficient oxygen channels for coal oxidation, the active groups in coal are also activated, and the secondary groups of active users continue to oxidize, thus the release of a large amount of heat. On the other hand, the thermal diffusivity and the thermal conductivity of the sample can be improved by the addition of mudstone, Lower its specific heat content, and support of thermal exchange of the coal sample^40^. The mudstone shows irregular heat absorption and release during the whole process.

#### Exothermic factor

The heat production of coal needs oxygen uptake, and the heat generation and oxygen uptake of the coal body lead to the oxygen partial in circumstances lower than that in the air. In the process of coal oxidation heating, the intensity of oxidation heat release is dominant, which is inextricably linked to the rate at which oxygen is consumed. Yan^45^ has studied the connection between the rate of oxygen uptake with the intensity of heat release of coal and has found that the exothermicity of coal is proportional to the rate of oxygen uptake, which can be expressed as:

6 $$HF=A{v}_{{{\text{o}}}_{2}}$$

where $A$ is defined as the exothermic factor, $HF$ is the exothermic intensity of each sample, and ${v}_{{{\text{o}}}_{2}}$ is the oxygen uptake rate of each sample.

**Figure 12:**
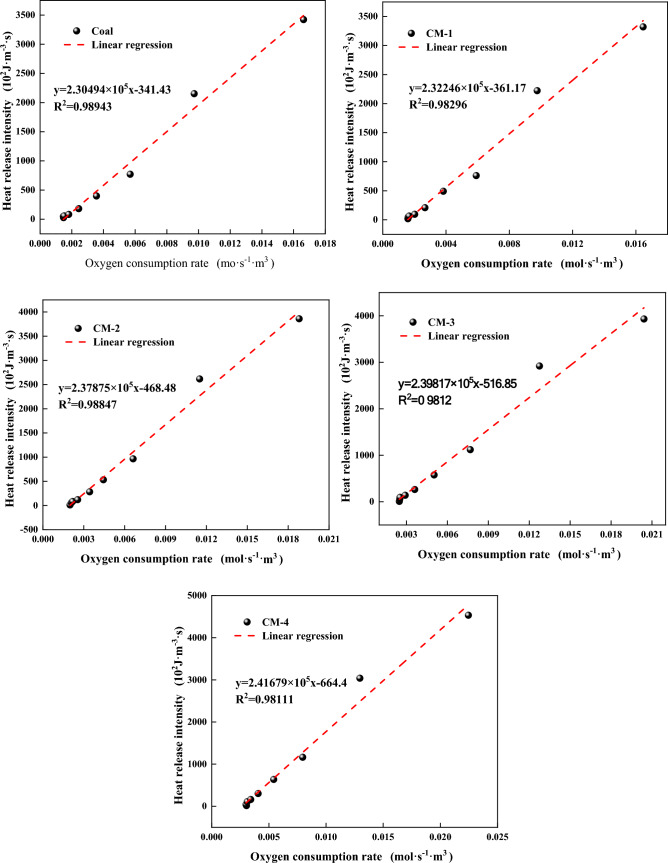
Fitting curve of exothermic factor.

The information from the exothermic intensity and oxygen uptake rate achieved by the fitting experiment of Eq. (6), is used to determine the relationship between the O_2_ consumed and the exothermic intensity at different oxidizing concentrations, see Fig. 12, and the fitting results in Fig. 12 are further counted, as shown in Table 3. The exothermic factor A of the samples added with mudstone increased by 0.01752–0.11185 relative to raw coal. The exothermic factor increased with the addition of more mudstone, and the two showed a linear relationship. It shows that the oxidation reaction of the sample after adding mudstone is more sensitive to the concentration of oxygen. The oxidation reaction rate of coal depends on the contact area between coal and oxygen to a certain extent. The addition of mudstone makes the specific surface area of the whole sample larger, the greater the surface area of contact between the dioxygen molecule and the carbon molecules, the more contact there is, and eventually the more severe the oxidation reaction. The exothermic factor A can be used as an index for the assessment of the severity of the oxidation effect of the self-ignition of coal samples. The exothermic factor of samples after adding mudstone is more than that of raw coal, implying that the risk of spontaneous combustion increases after adding mudstone.

**Table 3:** Comparison of exothermic factors of each sample.

Sample	A	R^2^
M	2.30494	0.98943
CM-1	2.32246	0.98296
CM-2	2.37875	0.98847
CM-3	2.39817	0.9812
CM-4	2.41679	0.98111

### Greenham fire coefficient

Graham observed that the gas produced after oxidation is related to adsorbed oxygen, and by comparing the rate at which CO is produced with the rate at which O_2_ is consumed, an index was established to calculate the level of heating^46,47^, which is still the most effective tool for detecting and evaluating underground fires in coal mines. This ratio is used to measure the oxidation strength of coal. In case of low temperatures, coal is oxidized to produce a small amount of carbon monoxide, while consuming a lot of oxygen. When the temperature rises, increasing amounts of oxygen are converted into carbon monoxide, so the ratio of the produced carbon monoxide to the removed or depleted oxygen will show strength. The defined fire coefficient is shown in Eq. (7):

7 $$k=\frac{\Delta CO}{\Delta {O}_{2}}\times 100\%$$

**Figure 13:**
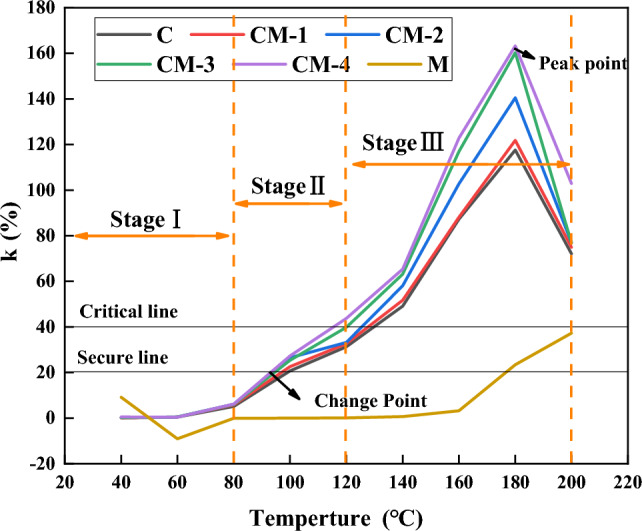
Variation of fire coefficient of each sample.

Figure 13 shows the variation of fire coefficient with temperature. It can be seen that the fire coefficient has a slight upward trend in stage one, but does not exceed the safety line. In the second stage, the change point begins to appear at about 100 °C. CM-4, CM-3, CM-2, CM-1, and C exceed the safety line in turn. At this time, active groups in coal and their high-energy compounds are destroyed, reacting with oxygen to release large amounts of gas. In stage III, with the increase of the addition amount, the critical line of 40% is exceeded, the temperature rises rapidly, reaches the ignition temperature quickly, reaches the peak at 180 °C, and the peak of CM-4 is up to 161%. It shows that the addition of mudstone makes the rate of increase of CO concentration and the rate of decrease of oxygen concentration change significantly, which accelerates the oxidation reaction, and the higher the mudstone content, the more obvious the oxidation acceleration effect.

### Activation energy analysis

According to the research of Zhong et al.^48,49^, the calculation formula of activation energy is shown in Eq. (8).

8 $${lnc}_{{\text{out}}}=-\frac{E}{R{T}_{{\text{i}}}}+{\text{ln}}(ASLm{c}_{{O}_{2}}^{{\text{n}}}/k{v}_{{\text{g}}})$$

where, $L$ is the length of the coal sample tank, m; ${c}_{{\text{out}}}$ is the CO concentration at the outlet of the coal sample tank. $S$ is the cross-sectional area of the coal sample container, m^2^; $k$ is the Unit Conversion Coefficient used to convert, 22.4 × 10^9^; ${v}_{{\text{g}}}$ is the air-flow rate, m^3^/s; ${T}_{i}$ is the absolute temperature of coal, K; $A$ is the pre-exponential factor; ${c}_{{O}_{2}}^{{\text{n}}}$ is the he content of oxygen in the reaction gas, mol/m^3^; $n$ is the order of reaction; $E$ is the energy-dissipating pile of activation, J/mol; $R$ is the molar gas constant, 8.314 J/(mol K).

**Figure 14:**
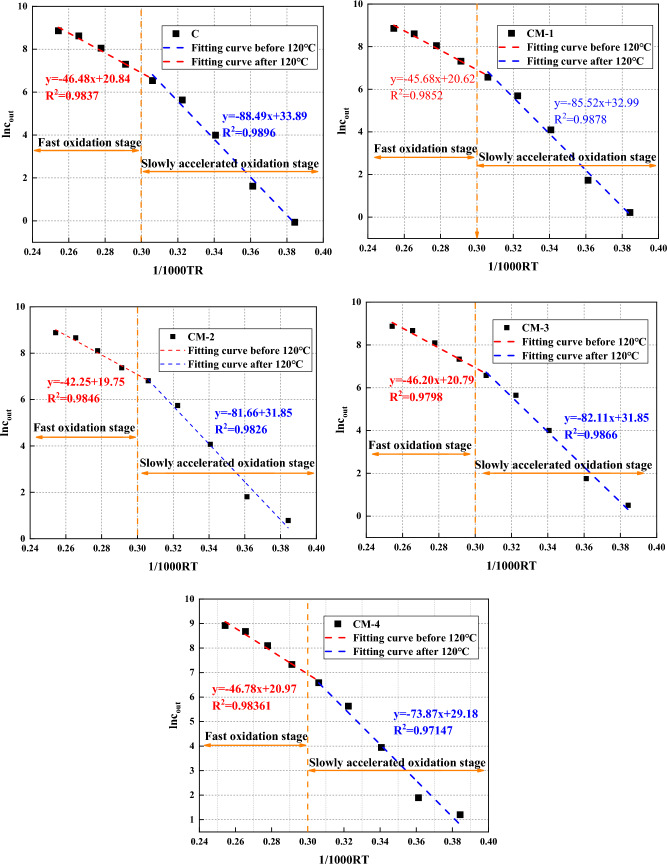
The results of apparent activation energy.

It can be seen from Eq. (8) that there is a linear relationship between ${ln}_{out}$ and 1/T when the ventilation flow rate is constant, and the equivalent activity of various stages of the coal-oxygen reaction can be obtained by computing the gradient. The LTO is divided into two processes. Before 120 °C, it belongs to the slow accelerated oxidation process, and after 120 °C, it belongs to the rapid oxidation process. The fitting calculation results are shown in Fig. 14. The changing trend of raw coal and mudstone samples in the whole LTO stage is the same, and the energy-dissipating pile of activation of the slow accelerated oxidation stage is always increasing than the appearance of the activity level of the rapid oxidation stage. At this stage, the energetic functional groups in coal rarely react with oxygen molecules, and more energy is needed to accelerate the oxidation of coal. In the phase of fast oxidation, with the increase of temperature, some inactive functional groups are transformed into active states, resulting in massive heat released during the coal oxidation process, resulting in a decrease in the energy-dissipating pile of activation compared with the slowly accelerated oxidation periods, and the apparent energy-dissipating pile of activation of the coal oxygen reaction continues to decrease. The surface activation energy of mudstone samples is less than that of raw coal. The slow accelerated oxidation period is 2.97, 6.83, 6.38, 14.62 kJ/mol eliminated than that of raw coal, and the rapid oxidation period is 0.28–4.23 kJ/mol less than raw coal. It shows that each stage is more prone to oxidation after adding mudstone, which makes the self-ignition tendency of coal lower.

### The mechanism of the effect of adding mudstone on the LTO stage of coal

**Figure 15:**
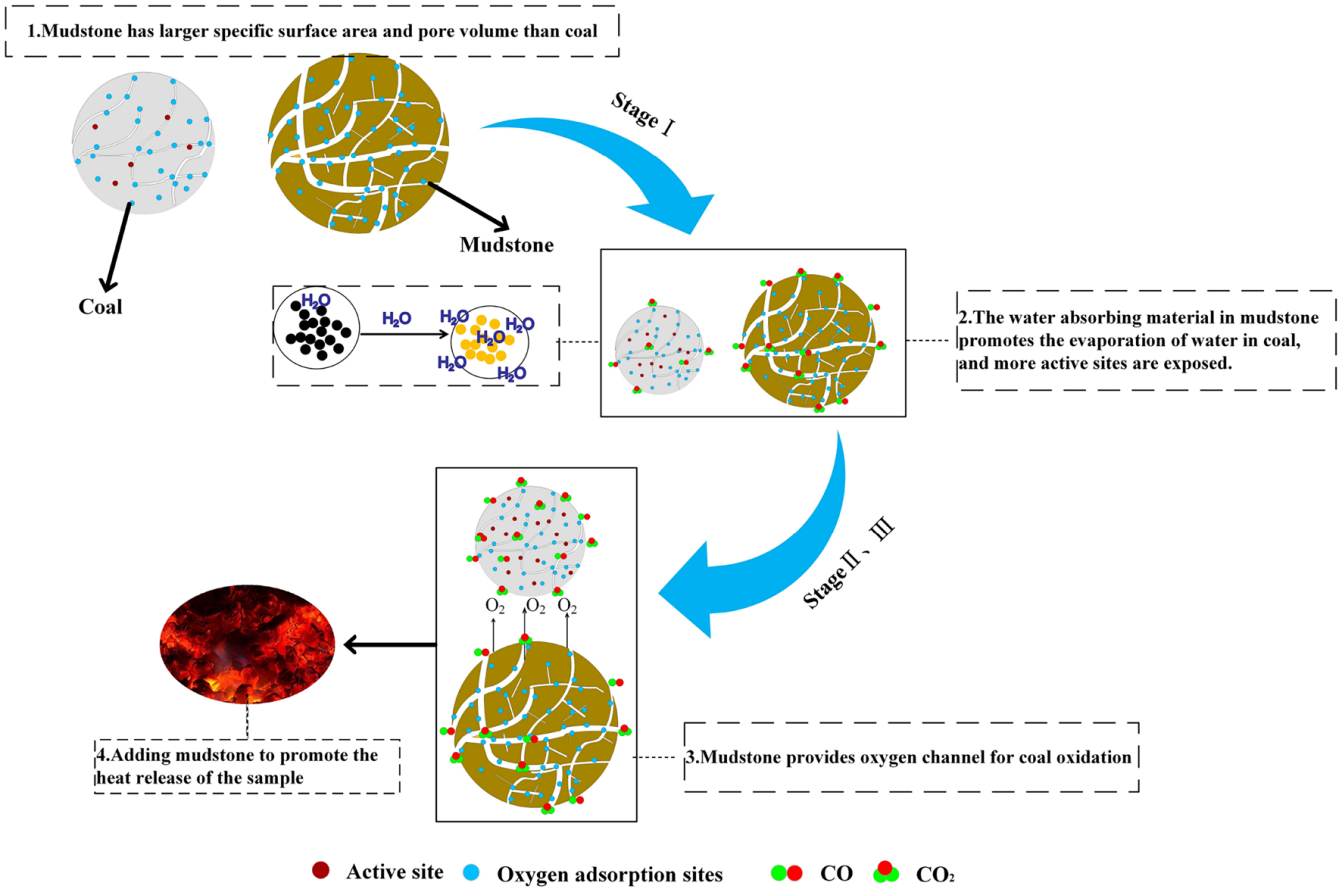
Mechanism of the effect of mudstone addition on the LTO process of coal.

To analyze the influence of mudstone on the LTO stage of coal spontaneous combustion, the pore structure characteristics of coal and mudstone, the physical and chemical parameters of the LTO experiment of coal-rock mixture with different mass ratios, and the changes of oxidation kinetic parameters were compared. Combined with the basic parameters of coal and mudstone, the mechanism of mudstone on the LTO of coal was determined, as shown in Fig. 15. There is an interaction between coal and mudstone, which is mainly based on mutual promotion. Adding mudstone accelerates its oxygen consumption, accelerates the heat release of the sample, reduces the energy required for its oxidation process, and achieves the effect of mutual promotion. In the first stage, the early stage of oxidation is dominated by water evaporation, because mudstone contains water-absorbing minerals, which accelerates the evaporation of water in coal, exposes active sites and pores and cracks of coal surface reacting with oxygen, and reaches the stage of accelerated and rapid oxidation (stages II and III). Mudstone has a larger specific surface area and pore volume than coal. The pore size distribution of mudstone is more uniform and the surface roughness is greater. These surface pores provide a good material channel for the transport of oxygen molecules, accelerate the oxidation of active sites, and increase the concentration of active centers. The increase of active center concentration and its continuous conversion with oxygen-containing functional groups leads to the accumulation of coal heat and the continuous increase of coal temperature. It promotes the exothermic chemical reaction and accelerates the oxidation reaction(The heat release intensity increases), accelerating the process of coal oxidation, thereby increasing the spontaneous combustion tendency of coal.

## Conclusion

Because of the effect of mudstone on the LTO process of coal, the physical parameters and oxidation kinetic parameters in the LTO process are analyzed by comparing the pore structure, specific surface area, and other related parameters of coal and mudstone. The main conclusions are as follows:

1. The specific surface area and pore volume of mudstone are 21.18 and 5.98 times that of coal, respectively. The pore size distribution of mudstone is more uniform. According to the fractal characteristics, the surface roughness of mudstone is larger and the pore structure is more complex, so mudstone can adsorb more gas.
2. With the increase of mudstone mass ratio, the amount of CO and CO_2_ produced by the sample increases slightly, and the oxygen consumption rate increases. According to the comparison of theoretical and actual gas products and oxygen consumption rate, the difference is linearly related to the content of mudstone added. The results show that there is a mutual promotion effect between mudstone and coal in the LTO process, and the more mudstone content, the more obvious the promotion effect.
3. The exothermic intensity of coal-rock mixtures with different addition ratios was calculated. The highest exothermic intensity of CM-4 was 4533.9 × 10^5^(J cm^−3^ s^−1^), which was 1.33 times that of raw coal. The exothermic factor A and Greenham fire coefficient k increased with the increase of mudstone content, indicating that CM-4 had the most obvious effect on the exothermic promotion of the coal LTO process. The apparent activation energy of mudstone samples with different proportions is 2.97–14.62 kJ/mol lower than that of raw coal, indicating that the oxidation reaction is more likely to occur in each stage after adding mudstone, which makes the spontaneous combustion tendency of coal lower. In summary, the addition of mudstone increases the spontaneous combustion tendency of coal in the LTO stage.

## Data Availability

The datasets used and/or analysed during the current study available from the corresponding author on reasonable request.
